# Population Data Centre Profile: SA NT DataLink (South Australia and Northern Territory)

**DOI:** 10.23889/ijpds.v4i2.1136

**Published:** 2019-12-05

**Authors:** M Schneider, CG Radbone, SA Vasquez, M Palfy, AK Stanley

**Affiliations:** 1 SA NT DataLink, GPO Box 2471 (CWE-49), Adelaide 5001, Australia

## Abstract

**Introduction:**

Originally piloted as a multi-departmental project within the Government of South Australia, SA NT DataLink is now the key provider of data linkage services in South Australia, the Northern Territory of Australia and the Commonwealth of Australia, enabling academics and policy makers to undertake research, policy, planning and evaluation.

**Approach:**

Uniquely governed by a broad range of consortium partners, SA NT DataLink’s business model operates with flexibility to adapt to researcher priorities and government requirements. Its Data Linkage Unit routinely links data from over 50 sources with more than 57 million records on approximately 2.9 million individuals. It arguably provides the broadest range of linked data sources in Australia, focusing on administrative datasets and clinical registries from various health and human services domains. Operating in strict adherence with the separation principal, SA NT DataLink’s Data Integration Unit separately manages anonymised clinical and service use data in collaboration with the respective data custodians through the Custodian Controlled Data Repository, allowing approved analysts to efficiently access high quality linked anonymised data. To protect individual privacy throughout the process of data linkage and data provision, SA NT DataLink’s processes align with state, territory and federal privacy legislations. Operating consistently with National Health and Medical Research Council guidelines, linkage projects are subject to approvals by the relevant data custodians and approved Human Research Ethics Committees.

**Noteworthy Outputs:**

SA NT DataLink has provided linkage services to over 160 data linkage projects, informing nationally significant research and policy initiatives, including initiatives to improve indigenous children’s hearing and child development.

**Conclusion:**

To respond to a changing data linkage landscape, SA NT DataLink is continuously reviewing and improving its systems, linkage processes and governance, addressing administrative, funding, data access, social licence and data linkage challenges and opportunities to meet increasing demand and new business developments.

## Article Structure

In the ‘Introduction’, the motivation to establish SA NT DataLink as well as its governance model is described. The ‘Approach’ section highlights the history, drivers and underpinning research leading to SA NT DataLink being the key data linkage unit for South Australia and the Northern Territory. The underlying sub-sections outline the administrative and data security processes (governance, consent and privacy models, data access), operational processes and procedures (population setting, operating model and data sources), linkage system and processes (linkage system, data linkage and linkage quality). The ‘Noteworthy Outputs’ section presents significant research projects using SA NT DataLink’s linkage services. DataLink’s current and future challenges, lessons learned and potential improvements are elaborated in the ‘Discussion’ while the ‘Conclusion’ recapitulates SA NT DataLink’s achievements as well as its role in the Australian data linkage landscape.

## Introduction

The importance of linkage data to improve health and wellbeing is well recognised [1]. Protecting individual privacy when sharing this information is vital, and SA NT DataLink [2] has been doing both for 10 years.

SA NT DataLink is the key provider of high-quality data linkage and data integration services for mobilising data from South Australia (SA) and the Northern Territory (NT). The data linkage system for SA and NT enables access to linked records for entire populations to allow more inclusive, representative, unbiased and cost-effective analysis. The organisation’s expertise is to link large volumes of unconnected and disparate information sources, including administratively collected data and clinical registries, to support a more informed evidence base for research, policy, planning and evaluation. As a member of the Population Health Research Network (PHRN) – a collaboration of data linkage facilities from across Australia that supports multi-jurisdictional and national linked data projects – SA NT DataLink facilitates access to approved information from within and across jurisdictions and data sources.

SA NT DataLink was established to provide access to a wide range of health, education and human services data, having as its basis the determinants of health. Data sources include hospital records, clinical registries, school data, public housing, homelessness, child protection and criminal justice data. Support is provided to enable additional linkage to Australian Government datasets such as the Medical Benefit Scheme (MBS) and Pharmaceutical Benefit Scheme (PBS) datasets, and, as the first pilot for Australia outside of Government, the Centrelink welfare and pension payments data.

SA NT DataLink is the operational body for a consortium formed under an unincorporated Joint Venture Agreement (JVA) between public universities, research organisation, government agencies and a consumer body in SA and NT. Consortium members provide both funding and in-kind contributions of time, effort and data for the successful operation of SA NT DataLink. The Australian Government provides financial support through the National Collaborative Research Infrastructure Strategy - Population Health Research Network (NCRIS PHRN).

Contrasting with most other Australian jurisdictional linkage units which are based within government agencies, this balance from a range of consortium partners contributing time and funding has allowed SA NT DataLink to be less reliant on government funding and develop priorities according to both researcher, non-government and government priorities.

## Approach

The need for reliable linkage services in SA was first clearly articulated by the research community and Government of South Australia in 2002, through the ‘Clients-in-Commons project’. This led to the linkage of records from several state government agencies, i.e. public hospitals, housing and community services, and the creation of an anonymised dataset of 1.5 million records for 410,000 clients allowing cross-agency analysis of service use and informing service provision and program development. While proving that mobilisation, standardisation and linkage of population data were possible and created valuable infrastructure for researchers and policy makers, this initiative also showed that the linkage process was labour-intensive and required a dedicated and skilled resource to allow infrastructure to be built and maintained to high quality standards in the long-term [3].

This initiative led to the development of a proposal for a population health and wellbeing data linkage system and a privacy protecting data linkage unit in South Australia. With the support of key government agencies such as the Department of Health and the Department of Education and the endorsement of the Privacy Committee of South Australia (the Privacy Committee), SA NT DataLink was established in 2009.

In response to increasing demand for data linkage services and with support from the Department of Health and the Privacy Committee, SA NT DataLink added the new business function of the Data Integration Unit (DIU) in 2018. The DIU manages anonymised clinical and service use records in a collaborative partnership with data custodians, who are the delegated authority responsible for considering and approving the release of data. The DIU operates a secure data repository, the Custodian Controlled Data Repository (CCDR), which has improved data quality and data delivery times for approved projects [4].

In 2017 SA NT DataLink was conditionally approved by the Australian Government as a Commonwealth Data Integrating Authority. Full accreditation as a Data Integrating Authority was granted in 2019, following regulation of SA NT DataLink under the Privacy Act, 1988 (Cth), which enables consideration of more efficient linkage services to Australian Government data (e.g. MBS and PBS).

SA NT DataLink’s organisational structure comprises four functional units, managed by its director, as illustrated in Figure 1 below.

**Figure 1: High-level overview of SA NT DataLink’s organisational structure, including responsibilities and full time employment equivalents (as of May 29th 2019):**
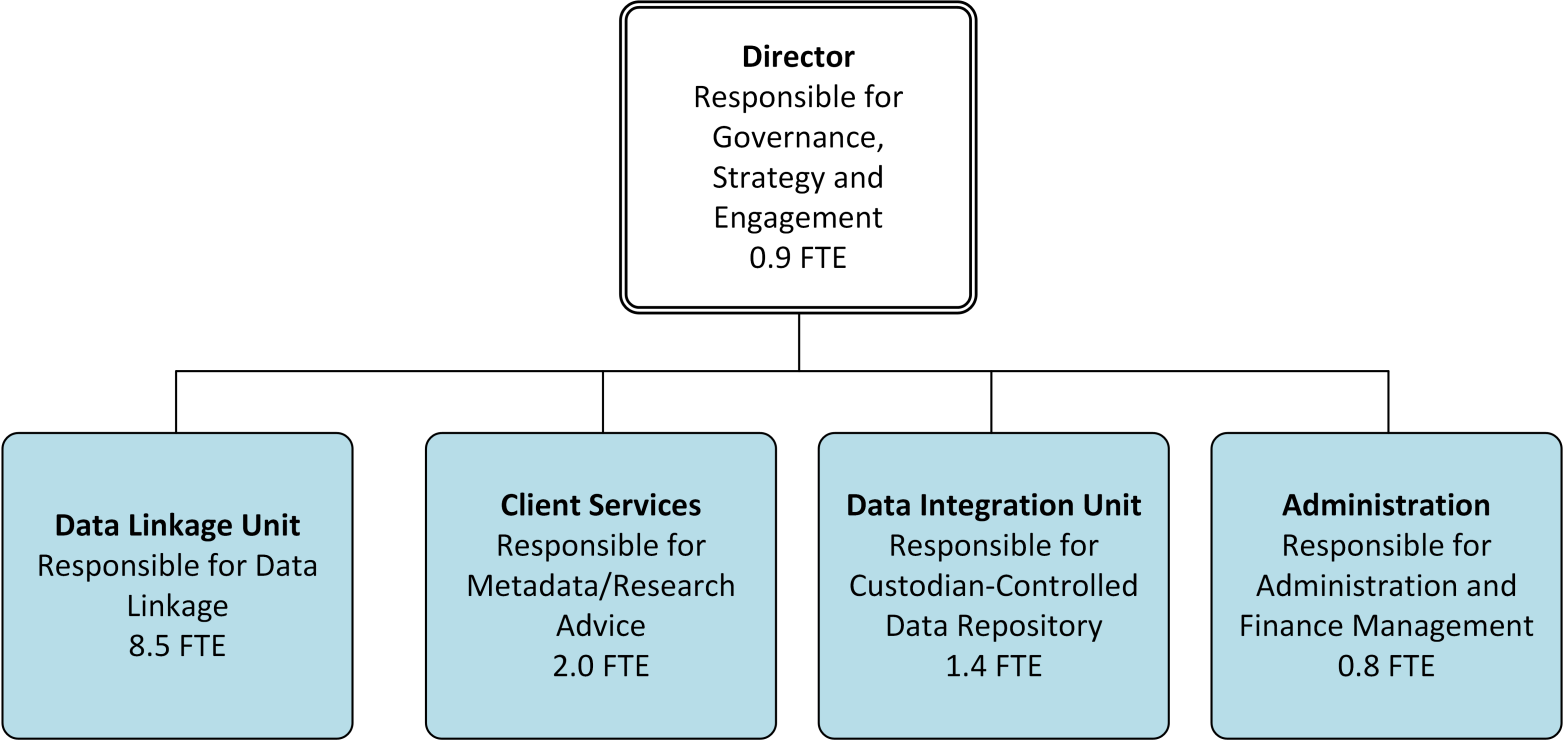


## Administration and security

### Governance, legislation and management

SA NT DataLink is governed by the JVA by which it was established. The JVA determines the joint venture partners and SA NT DataLink’s objectives, governance and operations as well as the duties, rights and obligations of the parties, which, in 2019, include:

- South Australian Government
- Northern Territory Government
- South Australian Health and Medical Research Institute (SAHMRI)
- Health Consumers Alliance of South Australia
- University of South Australia
- Flinders University
- The University of Adelaide
- Menzies School of Health Research, Charles Darwin University
- Cancer Council South Australia, the Beat Cancer Project

The Steering Committee is the peak governance body established under the JVA and is responsible for its overall policies and strategic objectives. The Director of SA NT DataLink is responsible for its day-to-day management and implementation of the Steering Committee’s objectives.

By agreement with the government agencies and the Privacy Committee, under the JVA, personally identifying data can only be accessed by approved personnel who must be in the employ of the South Australian Department for Health and Wellbeing. Therefore, this staff, as part of the public sector, are accountable for their actions to their employer under the *Public Sector Act, 2009 (SA)*. This employment arrangement is necessary to provide assurance to the data custodians and to the public, as this staff, who work on an air-gapped network isolated from any unsecured systems, in a physically separate and secure area of SA NT DataLink – the Data Linkage Unit (DLU) – access personally identifying information provided by government and non-government agencies. All other SA NT DataLink staff are employed by the University of South Australia, which acts as the administrating body on behalf of the joint venture (JV) partners managing the JV finances, risks and legal agreements. These University of South Australia staff members, including DIU staff operating anonymised data in the CCDR, cannot enter the DLU premises, unless supervised by DLU staff, and are therefore not able to access any personally identifying data.

While South Australia lacks specific privacy legislation, the SA NT DataLink operations must be consistent with the SA Government’s Information Privacy Principles (IPP) [5], which align with the Australian Privacy Principles (APP), legislated in the Australian Government’s *Privacy Act 1988 (Cth)*. The SA NT DataLink also operates consistently with the Northern Territory’s *Information Act 2002 (NT)*.

### Consent model

The provision of personally identifying information from government agencies to SA NT DataLink does not require consent from individuals for their data to be included into the SA NT DataLink system. Instead, an exemption to the IPP is granted through the South Australian Privacy Committee, established under State Government regulation and operating in the absence of jurisdictional privacy legislation. The Privacy Committee is responsible for approving the release of personally identifying information across the South Australian State Government and the Local Government jurisdictions. For the NT, the provision of personal information by data custodians to SA NT DataLink is governed by the jurisdictional privacy legislation, the *Information Act 2012 (NT)*. As privacy protecting infrastructure, no personally identifying information is released, except back to the originating data custodian for the purpose of their data quality improvement or when specifically directed by a data custodian. Separately, the *Public Sector (Data Sharing) Act 2016 (SA)* does enable the sharing of personally identifying information between government agencies in South Australia (including possibly SA NT DataLink) without the explicit consent of individuals. However, to date, SA NT DataLink has not provided such information under this act.

A waiver of consent for the provision of information on a case-by-case basis for each linkage project is permissible under section 95 of the *Privacy Act 1988 (Cth)*, which enables the provision of ‘information for research’ consistent with the National Health and Medical Research Council (NHMRC) guidelines. To authorise the use of data without the study cohort participants’ informed consent, the relevant NHMRC accredited Human Research Ethics Committee’s (HREC) approval is required as well as that of all data custodians whose data are being sought [6]. For consented studies, where individuals need to explicitly opt-in to be included or opt-out to be excluded from the research project, an ethics committee and data custodians assess the suggested consent model as well as the associated information material on individual consent [7].

### Privacy by design

**Figure 2: High-level diagram of the data linkage workflow and separation principle as it applies at SA NT DataLink (Source: SA NT DataLink).**
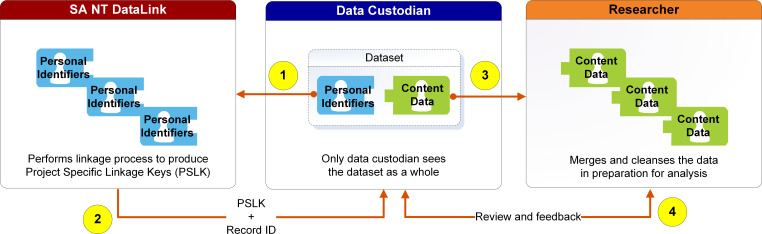


SA NT DataLink’s access to and provision of information is based on the separation principle, ensuring that information for data linkage is separated from information for data analysis [8]. This means that, apart from the data custodian, a person cannot have access to all of an individual’s data. DLU staff only access an individual’s personally identifying data, while approved persons analysing the content data only use the approved anonymised records (refer Figure 2 below).

The data linkage workflow illustrated in Figure 2 comprises the following steps:

1. The data custodian provides personally identifying data into SA NT DataLink.
2. SA NT DataLink produces Project Specific Linkage Keys (PSLK) and returns them to the data custodian.
3. The data custodian attaches PSLKs to anonymised content data and provides it to the researcher.
4. The researcher integrates and analyses anonymised content data from multiple custodians based on PSLKs.

Moreover, SA NT DataLink’s privacy protecting information and cyber security protocols align with the following security standards, to ensure the confidentiality, integrity and availability of the sensitive data:

- Australian Government ‘Protective Security Policy Framework’ [9]
- South Australian Information Security Management Framework [10]
- National Health and Medical Research Council (NHMRC) ‘Code for Responsible Conduct of Research’ [11]
- Population Health Research Network ‘Information Governance Framework’

Consistent with SA NT DataLink’s JVA, each data custodian providing data to SA NT DataLink signs a Data Agreement with SA NT DataLink, describing the conditions under which custodians supply data, the frequency of updates and how these data are stored, managed, linked, and on-provided by SA NT DataLink.

In addition, data custodians may require researchers to sign a specific Deed of Confidentiality governing the access, management and use of the data they have provided. Separately, all researchers must sign the SA NT DataLink ‘Researcher Deed of Confidentiality and Compliance’ governing access, management and use of all data they are provided with as well as their reporting requirements to SA NT DataLink and data custodians in relation to a breach of confidentiality.

### Data access

As stated previously, access to data for all research proposals supported by SA NT DataLink must be approved by the relevant HREC and data custodians. Once all governance approvals are in place, the linkage keys are generated and provided to the data custodians who then make the anonymised data available to the researcher.

In 2018, SA NT DataLink established its CCDR, operating a secure remotely accessible data repository and laboratory within the Secure Unified Research Environment (SURE) [12]. The CCDR is managed as a data safe haven by SA NT DataLink’s DIU. The CDDR allows custodians to provide anonymised datasets into the repository. These data are then reviewed for completeness and quality by the DIU in collaboration with the corresponding custodians. Datasets are held and maintained in the CCDR until approved by custodians for use in specific linkage projects and made available for an approved project by the DIU.

Data custodian approvals determine whether anonymised data are provided directly to the approved researchers, either by the custodian or via the CCDR, or made available for analysis in the CCDR. Where data from the Australian Government is used in a linkage project, these data are generally only accessible via a designated research workspace in SURE, such as the CCDR, also considered a data safe haven for Australian Government data.

## Operations

### Population setting

SA NT DataLink is the principal provider of data linkage services in the jurisdictions of South Australia and the Northern Territory, with respective populations of 1.74 million and 245,000 in 2018 [13].

Including historical records, SA NT DataLink holds more than 57 million unique transaction records within its Master Linkage File (MLF) on May 29th 2019, for approximately 2.9 million individuals in South Australia and the Northern Territory.

In addition to research studies undertaken on SA and NT records only, SA NT DataLink also provides data linkage services for SA and NT cohorts as part of multi-jurisdictional and national projects with data from other Australian states, the Australian Government or national registries. It also provides data quality review and geocoding address cleaning services to government agencies.

### Operating model

**Figure 3: Process steps of data linkage via SA NT DataLink (Source: SA NT DataLink):**
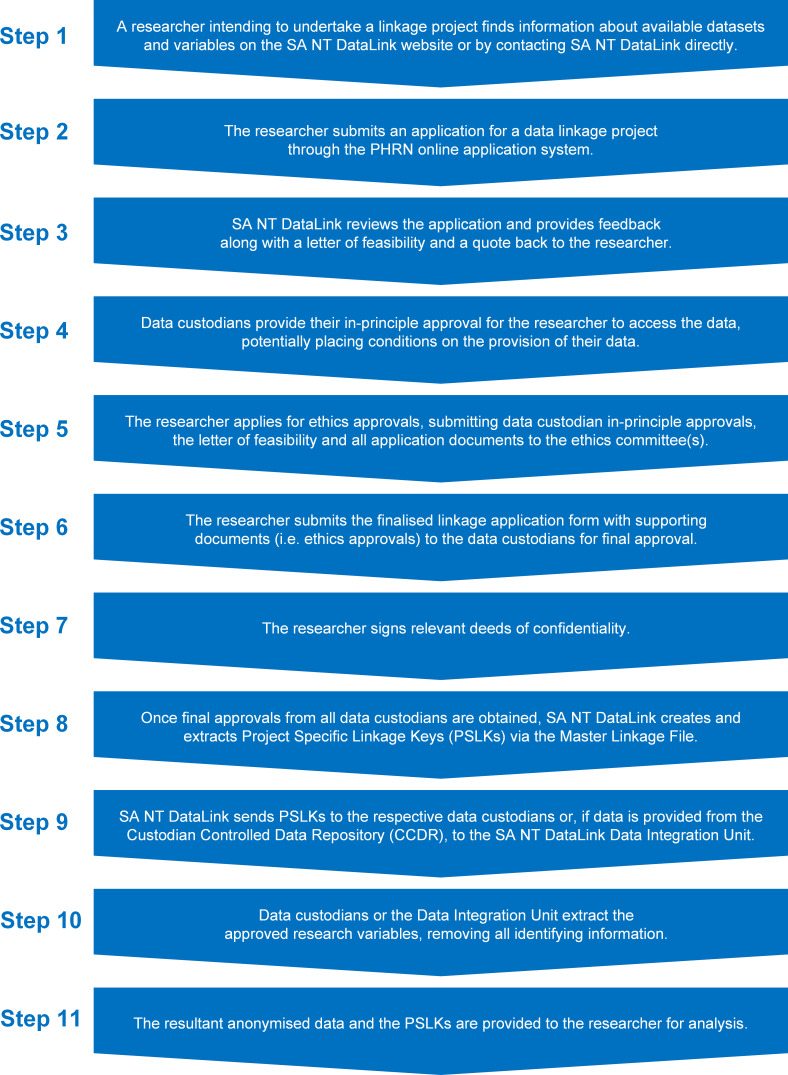


Figure 3 below illustrates the process of undertaking a data linkage project through SA NT DataLink, from the project initiation stage to the delivery of data to the researcher.

The anonymised clinical and service use data are currently stored in a distributed fashion by a multiple data custodians in various organisations and jurisdictions, as well as the CCDR. As outlined under step 10 (refer Figure 3 below), some anonymised datasets are available to approved researchers centrally via the CCDR while other data sources are provided directly by data custodians.

### Data sources

Table 1 below lists the name, temporal coverage, record count and number of individuals of datasets comprised in SA NT DataLink’s MLF in May 2019. It also indicates for which datasets the corresponding anonymised clinical and service use data is stored in the CCDR.

**Table 1: Names, temporal coverage and record counts for data sources available in the DLU and DIU (source: SA NT DataLink, May 29th 2019):** 

Dataset Name	Coverage	Number of Records	Number of Individuals	Anonymised content data stored in DIU

**Health Data**				
NT Client Master Index	1991-2017	4,224,874	854,222	
NT Perinatal (Trends) - by baby	1986-2016	108,731	108,689	
NT Perinatal (Trends) - by mother	1986-2016	104,754	62,837	
NT Inpatient Activity	2000-2017	242,693	201,167	
NT Primary Health Care Collection	2008-2017	94,557	73,743	
NT Emergency	2000-2017	438,140	316,028	
NT Public Hospital Pharmacy	2006-2014	Internally linked by NT Department of Health to its Client Master Index		
NT Hearing Health Data Collection	2008-2016	18,898	18,849	
SA Public Hospital Separations	2001-2018	7,112,477	1,404,479	Yes
SA Public Hospital Emergency Dept.	2003-2018	7,155,765	1,614,773	Yes
SA Dental Service (Titanium)	1999-2015	780,226	580,503	
SA Perinatal (linked by baby)	1986-2016	1,985,080	600,297	
SA Perinatal (linked by mother)	1986-2016	1,662,645	315,554	
SA Historic Cause of Death	1989-2005	195,930	195,930	Yes
SA Child Health Check	2005-2017	304,978	303,668	
SA Mental Health - Metro	1996-2017	625,550	258,652	
SA Mental Health - Country	2006-2015	128,265	128,265	
SA Drug and Alcohol Services	2002-2016	47,631	Not yet linked	
BreastScreen SA	2001-2018	384,453	339,015	
SA Cervical Screening	1993-2017	8,745,360	799,829	
**Education Data**				
NT Student Activity	2004-2018	474,863	96,272	
NT NAPLAN (Public Schools)	2008-2018	79,537	41,369	
NT Preschool	2005-2018	47,031	35,178	
NT NAPLAN (Catholic and Christian)	2008-2018	19,583	11,888	
SA Public School Enrolments Census	2004-2017	3,287,326	624,173	
SA NAPLAN	2008-2017	Internally linked by SA Department of Education		
SA Running Records	2008-2017			
SA English as an Additional Language	2005-2017			
SA Preschool	2009, 2012, 2015			
Australian Early Develop. Census	2009-2015	1,128,891	859,245	
**Registries**				
NT Birth Registry	1868-2018	184,201	183,965	
NT Death Registry	1870-2017	55,322	53,702	
NT Immunisation Register	1995-2018	436,443	288,031	
NT Rheumatic Heart Disease Register	1997-2017	4,078	4,078	
NT Cancer Registry	1991-2015	14,081	13,144	
SA Birth Registry (linked by baby)	1944-2019	1,498,252	1,427,152	Yes
SA Birth Registry (linked by mother)	1944-2019	2,599,835	1,065,295	Yes
SA Birth Registry (linked by father)	1944-2019	1,412,449	1,102,352	Yes
SA Death Registry	1990-2019	960,103	342,511	Yes
Codified Cause of Death SA	2006-2017	118,495	118,495	Yes
Codified Cause of Death NT	2006-2017	8,066	8,066	Yes
Coronial Codified Cause of Death SA	2006-2017	23,024	23,024	Yes
Coronial Codified Cause of Death NT	2006-2017	3,315	3,315	Yes
ANZData Registry	1963-2017	1,335,405	67,217	
SA Cancer Registry	1990-2016	497,116	214,958	
**Social Data**				
NT Child Protection	1998-2018	38,801	37,089	
NT Integrated Justice Information System (Adults)	1997-2018	115,260	10,319	
NT Integrated Justice Information System (Juveniles)	1997-2018			
NT Public Housing (Urban)	1991-2014	42,523	26,395	
SA Child Protection	1991-2017	587,575	213,276	
SA Electoral Roll	2009, 2013, 2017	3,380,007	1,390,983	
SA Youth Justice	1994-2016	14,710	13,827	
SA Homeless to Home	2011-2017	638,690	89,811	
Housing SA	1987-2017	3,926,272	1,020,660	

The datasets held in the DLU and DIU are updated annually, or as agreed with the respective data custodian required or as required for specific research projects. For example, record from the SA Public Hospital Separations and Emergency Department datasets are updated quarterly.

## Linkage system and processes

### Linkage System

Personally identifying data for linkage is stored in two separate database systems, both located on the same virtual server environment operating in an air-gapped network within the DLU, physically separate from unprotected systems. VMware server virtualization is used for hosting multiple virtual machines (VM) providing all the necessary services and storage needs. Up to 12 desktop clients in the DLU can connect to this server.

Data analysis and cleaning is undertaken using open source software (OSS), mainly R, and in-house developed tools, e.g. Cecilia [14], a R Shiny application. OSS is also used for linkage through a modified version of Freely Extensible Biomedical Record Linkage (FEBRL) [15], with added parallel processing and comparators [16]. The clerical review process relies on in-house software built around the Microsoft Office Suite, with a new version currently in development.

The current production system is based on MS SQL database running on a Windows Server VM. In this system, records are stored in tables together with grouping information, which identifies records for the same individual. The groups are derived from both deterministic and probabilistic linkage, using the record linkage software FEBRL, and the clerical review process.

Innovative linkage technologies and process improvements are being evaluated at SA NT Datalink. A selection of records have been loaded into the Next Generation Linkage Management System (NGLMS), which currently runs parallel to the MS SQL system. The NGLMS is based on open source technologies and platforms using a NoSQL graph database to store record information and, importantly, links and weights between them. Groups or clusters of related records can then be extracted using graph traversals. This makes the storage of links highly flexible, providing the ability to store all information about individual links and to add new links and relationships between records over time (e.g. for familial links [17]). Grouping of records on the same individual is not undertaken until the time of extraction. Moreover, this flexibility enables staff to choose the most suitable linkage for a study cohort. For example, if a project aims at maximising matches and can tolerate a certain level of false positives, a high sensitivity and low specificity project extraction strategy is applied.

The CCDR is operated in a private SURE workspace, which is a highly secure, remotely accessible computing environment, operated on data centres located in Australia. The data are managed in a PostgreSQL 10 database, which resides on a purpose-built database server within the SURE workspace. Data transfers into and out of the workspace are controlled by the SURE Curated Gateway, which is managed by the DIU to ensure unauthorised files cannot enter or leave the CCDR.

### Data Linkage

All datasets provided to the DLU undergo the initial step of de-duplication where deterministic linkage is applied across all records received from data custodians. Data are de-duplicated by assigning a unique group number indicating that the linkage variables for all records of the group are identical. Distinct header records, i.e. ‘representative’ records for the de-duplicated records, are then processed for probabilistic linkage against specific datasets or the whole MLF using FEBRL. The probabilistic linkage process results in record pairings, comprising variable-level comparisons. The linkage output is analysed, comparison scores are generated, and higher and lower thresholds are determined. Record pairs with comparison scores

- above the higher threshold are considered matches
- below the lower threshold are dismissed as non matches
- between the thresholds are treated as possible matches that are reviewed by the clerical review team, who decides whether to tag pairs as matches or as non matches.

Links for the confirmed matches are then loaded in a relational database, from which project extractions are undertaken. Master Linkage Keys (MLKs) are generated when records for new individuals are provided to the DLU causing new groups to be created. When additional records for an existing individual are received, those records are added to the same group under the existing MLK. MLKs are enduring links stored in the MLF and never released outside the DLU.

Extraction of data for a specific linkage project makes use of an algorithm that generates a PSLK for each study participant that will serve as their person specific identifier across all the datasets within this project. This ensures that data from different linkage projects potentially comprising the same individuals cannot be merged.

There is currently difficulty for state linkage units to receive personally identifying information from the Australian Government. Therefore, where Australian Government data are approved for a linkage project, all required SA datasets are firstly linked by the DLU, and the corresponding PSLKs and the personally identifying information are then sent to the Australian Institute for Health and Welfare (AIHW) data linkage unit. The AIHW undertakes the linkage for all relevant Australian Government datasets and makes the anonymised linked data accessible to researchers in SURE.

Where the study cohort has consented, no statistical disclosure controls are applied. For unconsented studies, data custodians may impose minimum cell size to manage the risk of re-identification. This could apply where individuals are likely to be re-identified, e.g. for sparsely populated areas.

### Linkage Quality

In addition to systematic clerical review of probabilistic linkage outputs, based on the comparison scores between the lower and higher thresholds, the following quality assurance activities are carried out to identify and review groups of records that may contain false links, either false positives or false negatives.

- Before the loading of data for linkage, logic checks are undertaken to ascertain the accuracy and completeness of the received data. This process has been automated for all incoming data using Cecilia, which also normalises the records before linkage. An example of a logic check is to test for any activity after the date of death or prior to birth.
- There are also business rules to verify the integrity of the MLF and assess the linkage results. For example, the ratio of MLKs to patient identifiers is calculated since there should only be one MLK per patient identifier.
- For each project, a series of quality checks is undertaken on the linkage outputs, following a standard developed through a collaboration with other Australian data linkage units and the PHRN.
- Where required, targeted manual clerical review of linkage outputs is undertaken.

### Noteworthy outputs

The following paragraphs highlight key projects that have helped identify health or social problems and improve the lives of individuals and communities in Australia.

From 2010 to 2014, The ‘NT Early Childhood Development Demonstration Project - Improving Early Child Development and Educational Outcomes in the NT’ has employed linkage data from SA NT DataLink. Used data sources include birth and immunisation records, the Australian Early Development Census (AEDC), and school enrolment, attendance and exam results for approximately 60,000 NT children for the NT population, 40% of which are indigenous. Key findings [18, 19] include the following results:

- The project highlights the extent to which sociocultural and economic circumstances influence all children’s early health, development and learning;
- Addressing community-level factors, such as housing overcrowding, is likely to result in substantial improvements in school attendance, especially in very remote communities;
- Improving levels of attendance at preschool offers one of the best immediately available strategies for improving the NT’s rates of Aboriginal school attendance and achievement.

A follow-up project, effectively a research program, the ‘Developmental Outcomes for Children and Youth in the NT’ also solicited SA NT DataLink’s linkage services. This project assessed disadvantage experienced in indigenous communities, providing an empirical evidence base for the influences on health and wellbeing, education, child protection and youth justice outcomes. A sub-study provided essential data to the Royal Commission into the Protection and Detention of Children in the Northern Territory. Another sub-study that generated interest was the issue of poor hearing health in Aboriginal children and their trajectory through the education, employment and criminal justice systems. The sub-study showed how the Aboriginal children’s hearing loss, which is preventable, negatively impacts their life course. Following this, the Australian Government Department of the Prime Minister and Cabinet announced in August 2018 additional resources of $8 million over five years for the ‘Hearing Health Learning Initiative’. This funding is targeted at on ground intervention and health services to improve indigenous children’s hearing and to support improve their life outcomes.

The South Australian Early Childhood Data Project (ECDP) has also used SA NT DataLink’s data linkage services. This project is a program of research using early childhood, birth, death, hospital, housing, child protection, Australian Early Development Census, school and justice data, and has established one of the most comprehensive population-based research databases in Australia. The initial linking of 15 data sources has been expanded to now providing access to 27 data sources from health, education and welfare services. It has enabled the investigation of a wide spectrum of child health and development outcomes. This work led to the creation of a significant ‘public good longitudinal research resource for South Australia’, with research partners in multiple disciplines and sectors in academia and government. This has translated research into policy impact helping to advise government and non-government sectors [20].

## Discussion

### Governance

Unlike other linkage units in Australia, SA NT DataLink is not embedded in a government department but is governed by a consortium of multiple partners, including universities, research organisations and government agencies. This makes SA NT DataLink less reliable on a single organisation’s priorities, balancing strategic directions through building infrastructure that meets a combination of researcher, non-government and government needs.

### Data Sources

SA NT DataLink was the first non-government agency approved as a Commonwealth Data Integrating Authority, in November 2017, conditional on privacy legislative coverage. This has been confirmed by its full accreditation in 2019. SA NT DataLink enables researchers to access arguably the broadest range of data sources available in Australia, including health, education, criminal justice and human services datasets. Integrating data from the government and private sector, e.g. private health service and public hospital data, would further improve its utility and allow researchers to gain a more comprehensive understanding of population-wide trends and service outcomes. While public sources of data are increasingly being made available for linkage purposes via SA NT DataLink, the acquisition of private and non-government datasets has proven to be protracted. Where data access and use agreements with government or non-government are in place, the subsequent provision of data for approved projects is straightforward.

Even where data is made accessible, there can still be a significant delay between the time of data collection and its use for analysis. Furthermore, there are resource constraints and data quality and completeness issues. In response to an increasing demand from researchers and analysts, and their expectations for more timely delivery, the automation of processes, including approvals and provisioning, is essential.

Researchers and analysts are increasingly seeking access to wide range of data sources, often from diverging domains. Organising access to these data and the corresponding data governance often requires considerable time to refine the proposals for approval. Moreover, due to the lack of standardised data and metadata collections or exchange protocols, interoperability frameworks and standards are urgently required. This includes common ontologies to harmonise data structures, terminology, coding and quality. SA NT DataLink endeavours to make datasets held in the CCDR compatible with the Fast Healthcare Interoperability Resources (FHIR) specification [21], the industry standard for healthcare data exchange. FHIR applies SNOMED Clinical Terms [22] to harmonise medical terms, codes, synonyms and definitions.

Ideally, an integrated cross-institutional data sharing and analysis capability would support innovative research.

### Business opportunities

Access to near real-time data could also create opportunities for new business activities under the current privacy protecting model, possibly integrating government and non-government data. These innovative data integration services could help inform health services improvement by integrating across a range of human services.

Thanks to its broad governance structure and technical expertise, SA NT DataLink is well positioned to collaborate on innovative data driven research projects and infrastructure initiatives. Currently, SA NT DataLink supports the growing focus on machine learning, precision health and genomics, in response to an ageing population, skills shortage and economic constraints.

As an additional business development opportunity, SA NT DataLink can expand on its role as a trusted third party, providing value by managing unique cross-agency and organisation-wide identifiers for improved data quality and use.

### Social Licence

Engagement with the community and consumers to foster confidence that their privacy is being protected is fundamental to SA NT DataLink’s operation. Due to strict data security protocols, SA NT DataLink has not experienced any major accidental and intentional data security incidents in its 10 years of operation. Multiple major data security breaches in private companies and through government have compromised the social licence and diminished public trust into the ability of organisations to protect privacy. To maintain the social license, significant investment into data security technologies and cyber security practices are required to minimise risks and to demonstrate the ongoing commitment to protecting privacy.

### Data Linkage

The lack of consistent personal identifiers in Australia is a key reason why the current data linkage model exist. In Australia, there have been lost opportunities to embed the ability to share records for policy, planning, evaluation and research into legislation. For example, the *Australia Card Bill 1986 (Cth)* for a citizen centric identifier, introduced in the Australian Parliament 33 years ago, would have better positioned Australia to legally share records. In recent years, there have been initiatives that have resulted in the improved and secure sharing of digital health records. However, they have focussed on discrete domains of government-funded activity. For example, the *Healthcare Identifiers Act 2010 (Cth)* is used by authorised parties to enable electronic health records to be effectively shared, but this exists separately from national individual identifiers schemes. The lack of a shared and common citizen identifier is the principle reason for the continuing challenge to share data across domains of activity in Australia.

### Demand

**Figure 4: Process steps of data linkage via SA NT DataLink (Source: SA NT DataLink):**
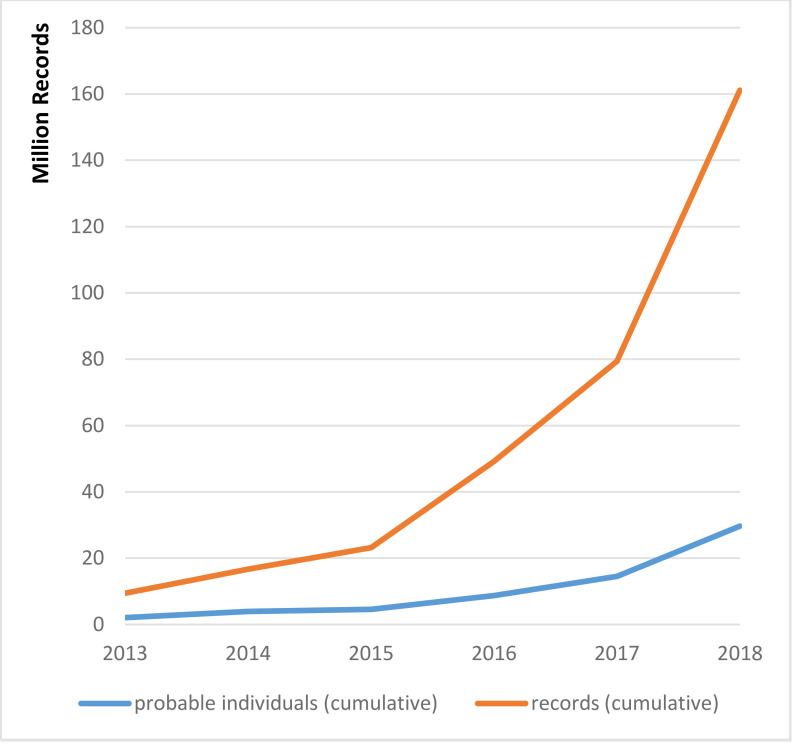


Since 2013, the growth in the demand for data linkage services has significantly increased (refer Figure 4 below), with individual studies also comprising increasing numbers of records and individuals.

Since this trending growth is expected to continue, SA NT DataLink is striving to improve the efficiency of the entire data linkage project lifecycle, from project application to data delivery and tracking of outcomes, by:

- automating the transfer, quality review and loading of data from data custodians via secure automated extract-transform-load (ETL) tools and Cecilia (data cleaning and pre-loading tool);
- reducing the time and effort required to complete clerical review and to add new linked records to the MLF while maintaining linkage quality by implementing the learnings and process improvements from the NGLMS;
- expanding the data holdings in the CCDR to improve data quality and reduce delivery times of anonymised content data to approved researchers;
- receiving unconditional approval as a Commonwealth Data Integrating Authority, allowing SA NT DataLink to become a trusted party able to link Australian Government datasets, such as MBS, PBS and immigration data; enabling SA NT DataLink to undertake all required linkage will significantly decrease delivery times and increase linkage quality, including those involving Australian Government data.
- developing a project tracking and status reporting tool to improve productivity in client services, DLU and DIU;
- providing more comprehensive metadata for SA NT DataLink’s data holdings to researchers to facilitate the selection of data variables during the project initiation stage.

## Conclusion

The high quality linkage service at SA NT DataLink has a focus on the broad determinants of health, supporting evidence based research and analysis on themes ranging from early childhood development, child abuse and neglect to cancer and cardiac treatment outcomes and indigenous health and wellbeing. SA NT DataLink is well positioned to be responsive to both government and research priorities, supporting innovative access and privacy protected analysis of people’s records across health, education and human services. As the authorised data linkage unit responsible for SA and NT, SA NT DataLink has successfully provided linkage services for over 160 projects and programs of research over the 10 years of its operation. This has enabled academics to undertake innovative research projects and assisted government planning and evaluation of policies and programs, gaining novel insight into population-wide trends and individual outcomes. Royal Commissions conducted into child abuse and neglect as well as aged care services across Australia have relied on data provided through SA NT DataLink. Importantly, these linkage projects have contributed to important policy changes, making a real difference to improve people’s lives.
